# GPs asking patients to self-rate their health: a qualitative study

**DOI:** 10.3399/bjgp15X686557

**Published:** 2015-09-01

**Authors:** Göran Waller, Katarina Hamberg, Annika Forssén

**Affiliations:** Department of Public Health and Clinical Medicine, Division of Family Medicine, Umeå University, Umeå, Sweden.; Department of Public Health and Clinical Medicine, Division of Family Medicine, Umeå University, Umeå, Sweden.; Department of Public Health and Clinical Medicine, Division of Family Medicine, Umeå University, Umeå, Sweden.

**Keywords:** comparative self-rated health, consultations, family medicine, GP, qualitative analysis

## Abstract

**Background:**

In epidemiological research, self-rated health is an independent predictor of mortality, cardiovascular diseases, and other critical outcomes. It is recommended for clinical use, but research is lacking.

**Aim:**

To investigate what happens in consultations when the question ‘How would you assess your general health compared with others your own age?’ is posed.

**Design and setting:**

Authentic consultations with GPs at health centres in Sweden.

**Method:**

Thirty-three planned visits concerning diabetes, pain, or undiagnosed symptoms were voice-recorded. Dialogue regarding self-rated health was transcribed verbatim and analysed using a systematic text condensation method. Speaking time of patients and doctors was measured and the doctors’ assessment of the value of the question was documented in a short questionnaire.

**Results:**

Two overarching themes are used to describe patients’ responses to the question. First, there was an immediate reaction, often expressing strong emotions, setting the tone of the dialogue and influencing the continued conversation. This was followed by reflection regarding their functional ability, management of illnesses and risks, and/or situation in life. The GPs maintained an attitude of active listening. They sometimes reported a slight increase in consultation time or feeling disturbed by the question, but mostly judged it as valuable, shedding additional light on the patients’ situation and making it easier to discuss difficulties and resources. The patients’ speaking time increased noticeably during this part of the consultation.

**Conclusion:**

Asking patients to comparatively self-rate their health is an effective tool in general practice.

## INTRODUCTION

In epidemiology, self-rated health is measured as an individual’s subjective answer to a question about their general health (global question) or their health compared with others their own age (comparative question), and has a strong association with significant medical outcomes such as death, diabetes, coronary heart disease, functional ability, and depression.^1^,^2^ Self-rated health is also associated with: age, education, social capital, pain, functional status, low spirits, and medical diagnoses, and how these are perceived.^2^

A seminal review and several epidemiological studies recommend self-rated health for clinical use.^2^–^7^ To the authors’ knowledge, however, research regarding such use is sparse. Only one study has been retrieved concerning actual use of self-rated health in a clinical setting.^8^ Two GPs asked patients to estimate their self-rated health on a scale of 0 to 100. Patients with ratings >50 were asked how they, despite obvious medical problems, could maintain a positive self-rating. The authors concluded that the doctor can contribute to patient empowerment by exploring and recognising the patient’s view of their general health, and that such knowledge is at the core of clinical practice.^8^

The formulation of the question is of minor importance in relation to outcomes such as mortality in epidemiological studies.^9^ Age-comparative and global questions of self-rated health are different, however, and elicit different response patterns.^10^–^12^ When comparing the two questions semantically, the comparative question was found to be clearer than the global one, particularly as it gives a frame of reference for the assessment.^12^

This study aimed to investigate what happens when the question ‘How would you assess your general health compared with others your own age? Is it better, about the same, or worse?’ is posed to patients in authentic consultations with GPs.

## METHOD

Eight experienced colleagues interested in consultation skills were invited to participate. Six accepted, and two of the authors also participated. The participants received an outline of the study design and brought a recorded pilot consultation to an introductory meeting with all participating physicians. At that meeting, the epidemiological background of self-rated health and the aim of the study were presented, and discussions were held on how to perform the study. The question was to be posed when convenient before the physical examination; the exact wording could be varied, but the words ‘assess’, ‘health’, and ‘compare with others your own age’ were important. Emphasis was not to be on pinpointing comparisons, but on attentive listening to the patients.

The consultations were scheduled, non-emergency appointments with new as well as established patients. In keeping with previous research, patients with diabetes mellitus type 2 and chronic non-malignant pain were chosen, adding also patients with undiagnosed symptoms. Patients were aged ≥18 years and able to speak Swedish. The aim was to obtain a wide age spectrum and a balanced number of females and males, accomplished through feedback to the participating GPs. No further selection was deemed necessary. Thirty-three consultations were judged to be sufficient; the last recordings did not change the main findings of the study.

**How this fits in**

Knowledge of self-rated health is based on epidemiological studies. Self-rated health is a comprehensive summary of several factors important for health, it is predictive of critical medical outcomes, and has been recommended as a clinical tool. The application of this epidemiological concept to clinical situations has scarcely been studied. This qualitative study reports what happened in authentic consultations in general practice when patients were asked to self-rate their health.

Patients were informed that the study concerned how doctors can improve dialogue with patients. All patients gave written informed consent to participate.

The consultations were voicerecorded. One author listed topics covered in each consultation and established which portions concerned the question. Consultation time as a whole and the overall apportionment of speaking time between doctor and patient were measured and specifically during the discussion of self-rated health. Time used for administrative tasks such as writing prescriptions was not included. Decisions on how to delineate topics, and that pauses >1 second were signs of cognitive processes, were informed by the Roter interaction analysis system.^13^ The first 10 consultations were transcribed in full. Otherwise only the discussion of self-rated health was transcribed.

The authors separately made a written summary of the portion of each transcript devoted to the question. Further analysis was informed by the method of systematic text condensation (STC).^14^ The quotations were edited slightly to make them readable.^15^

Immediately after each consultation, the doctors completed a questionnaire asking: ‘Did the question of self-rated health affect the consultation and/or your understanding of the patient’s health situation, yes or no? Describe how’. The answers to the ‘describe how’ question were used in the final analysis as a means of mirroring the STC analysis of the transcriptions.

## RESULTS

### Participants and speaking time

There were 33 participants in the study: 17 females and 16 males, aged 18–83 years, median age 60 years.

Reasons for consulting are listed in Table 1. ‘Other’ reasons included stomach problems, worries about heart disease, headache, dizziness, lung disease, exhaustion, and weight problems.

**Table 1.** Reasons for consultation

**Main reason**	**Females, *n***	**Males, *n***	**Total, *n***
Diabetes	7	9	16
Pain	3	2	5
Other	7	5	12
Total	17	16	33

The participating GPs, three males and five females, were aged 44–61 years and had been working as physicians for 16–34 years. The consultations took place between May 2013 and November 2013 at community health centres in northern Sweden, located in towns as well as small municipalities.

Time used for the consultations ranged from 12 minutes to 46 minutes (median 23 minutes). Time for discussion of self-rated health ranged from 30 seconds to 15 minutes, (median 2 minutes). In the consultation as a whole, the patients’ speaking time varied from 21% to 85% of the total speaking time. In the portions covering self-rated health, patients’ speaking time ranged from 49% to 90%. The increase was most prominent in the consultation where the patient had only 21% of the total speaking time. When discussing self-rated health, the patient’s speaking time rose to 64%. Generally, the apportionment of time was associated with the doctor’s consultation style.

### An unfamiliar and unexpected question

Physicians stated that the question had affected the consultation and/or their understanding of a patient’s health condition in 30 of 33 consultations. On two occasions, incorporating the question and/or being recorded was experienced as distracting. On two other occasions, the question had made the consultation slightly longer. The remaining comments are integrated into the results below.

Doctors did not always use the comparative words ‘better’, ‘worse’, or ‘about the same’, and even if they did the question did not always elicit a distinct answer. In total, 12 patients indicated that their health was ‘worse’ than others in their age group, 13 replied ‘better’, and eight ‘about the same’.

The question was often followed by silence lasting several seconds; some patients sighed or made a tentative attempt to respond; some asked the physician to repeat the question. In response to such confusion, physicians sometimes made the question more transparent by providing the three alternative answers.

This process suggests that the question was unexpected. Once patients had understood it, many responded emotionally (referred to below as ‘reaction’), and subsequently more thoughtfully (referred to as ‘reflection’).

### Reaction

Many patients interpreted the question as referring to lifestyle matters — diet, weight, and exercise — and for most this brought on feelings of guilt and shame. One such immediate response was:

*‘Well, I do have a spare tyre.’* [embarrassed laugh]

(Male, 65 years, diabetes check-up)

When patients conveyed guilt and shame the physicians reported that they became more sensitive in discussions of lifestyle issues. Some patients, in contrast, reacted with pride and delight, asserting that they were in better shape than others. The physicians experienced such responses as ‘lightening things up’.

Another emotion that came out was grief, sometimes expressed as anger, about poor health and the limitations this entailed, or being so much worse than others. These feelings were often expressed in strong language, whereas profanity did not otherwise occur in consultations. One patient burst out:

‘Some people just feel so damned good!’

(Male, 71 years, chronic fatigue, stomach problems)

Even with established patients, a powerful emotional reaction could make the physician view their situation more clearly: ‘Things were worse than I’d realised’. Other reactions provided entirely new insights; debilitating symptoms that the physician did not know about; no peer group for comparison revealed isolation despite an apparently normal social life.

However, there were also patients who stated that they felt pretty good, despite their current symptoms. Even a serious chronic illness might sometimes, in comparison, seem tolerable:

‘I don’t know, there’s sure to be someone who’s in even worse shape than I am’.

(Female, 53 years, severely disabled by rheumatoid arthritis)

According to the physicians, the emotional responses gave an on-the-spot account of the patient’s situation and had an impact on the atmosphere in the room and the direction of follow-up discussion.

### Reflection

In discussions that followed, focus was on the patients’ thoughts and reflections about their health, past and present. They expressed insights that seemed to evolve or coalesce during the conversation. The physicians responded with encouraging murmurs and by repeating or summarising what the patients had just said, making it easier for them to continue. The conversations focused on the patients’ functional ability, their ways of managing symptoms, illnesses, and risks, and how their life circumstances affected their experience of ill health.

#### Functional ability

Illnesses and symptoms were clearly correlated with functional ability in daily life after the question was posed. Some patients thought they felt better than before, physically, emotionally, or in general. Others began identifying what they were able to do despite everything, and things they could enjoy and afford. Comparisons with others who were more limited by illnesses made some look more positively on their own situation, even those who initially had answered ‘worse’. An 83-year-old male who no longer could go hunting because of difficulty walking was one of these. When, towards the end of the consultation, the doctor returned to the question, the patient responded:

Patient: *‘As I said at the beginning, I’m a bit disabled because of my legs.*’

GP: ‘Yes, right.’

Patient: ‘Don’t have much strength ...’

GP: ‘Mmm.’

Patient: ‘My balance is worse, and … But other than that, I think overall I’m doing better than many … A lot of others are dead and … I’m clear in the head, after all.’

This type of shift in perspective made it easier to discuss the patient’s resources. As one physician wrote: ‘It stimulated me to try to inspire change’.

However, for other patients illness and its negative consequences continued to be the central focus. Such exchanges could give physicians a deeper awareness of the patient’s problem, insight into the gravity of the illness, or the risk of continued ill health. On rare occasions, physicians reported that the question only made the patients ‘start thinking even more about how miserable they were’.

#### Managing symptoms, illnesses, and risks

The patients also considered how they attempted to manage their ill health. Many described having begun, after great struggle and internal resistance, to accept their situation: ageing was inevitable, illnesses required medication, and they had to make the best of it. Reflections like this incorporated a degree of detachment or self-irony, and occasionally the tone became joking.

Work and taking responsibility were among the resources and deliberate strategies that patients brought up. One retired patient helping a friend at his firm stated that this made him ‘forget his troubles for a little while’. A female suffering from chronic pain and agoraphobia after being raped described having realised that she had to face up to her fears for her child’s sake:

Patient: ‘… when I realised that my son and I never could go to the movies on our own, or go shopping or walk around town on our own …’

GP: ‘Mmm.’

Patient: ‘… well, that’s when I pulled myself together and ... started, what do you call it, working through it.’

The physicians commented that conversations like this made both participants aware of the obstacles the patient had dealt with. Other patients emphasised, in ways the physicians had not anticipated, being ‘bull-headed’ in their determination to overcome physical disabilities. These attitudes could be useful when discussing treatment.

Many patients emphasised their attempts to ‘live a more healthy life’, and some had succeeded. Others were well aware of what they needed to do but failed continuously. The burden of responsibility and inability to live up to expectations weighed heavily on them. A 60-year-old male, worn down by multiple illnesses and answering ‘worse’ had lost his ‘go’:

Patient: ‘They say I have to eat the right food, I have to exercise, I have to blah blah blah.’

GP: ‘Mmm.’

Patient: ‘I was supposed to lose some weight, but I haven’t … You know, I just don’t get around to it … it’s just that it’s so hard to deal with ...’

When patients could express their sense of futility aloud, physicians felt it became easier to provide support.

#### Life circumstances

The patient’s circumstances in life and their impact, for better or worse, on symptoms and illness came up in many discussions. A 71-year-old male began reflecting on his isolation:

‘Well, I’ve been sitting and thinking about this, if it could be something like … having too little to do … that I spend too much time alone, and then, well, I brood about it consciously, or brood about it unconsciously, about my situation, I mean the way things are, you see.’

Several females described difficulties in relationships with partners. One female was not taking her painkillers because her husband made disdainful comments about her dependence on them. Another female with chronic pain enjoyed being at the stable with her daughter, but her pleasure was diminished because her husband ‘wasn’t so crazy about it’.

Patient: *‘Well, it’s like he feels cut off or something.’* [laughs]

GP: ‘I see.’

Patient: ‘He’s like, “Oh, are you off to the stable again? When will you be back?”’

GP: ‘Mmm.’

Patient: ‘… it’s not as much fun.’

The patients’ reflections about their life circumstances gave the physicians a more complete picture of them as individuals and the challenges they faced.

## DISCUSSION

### Summary

In most consultations, asking patients to comparatively self-rate their health had an impact, including on the tone of the discussion. The patients’ speaking time increased, while the physicians’ role shifted to encouraging them to talk. The first reaction to the question, often spontaneously emotional, was followed by a reflective discussion in which patients weighed various reasons for their self-assessment. These reflections gave a telling description of the patients’ functional ability, life circumstances, and resources for or obstacles to managing symptoms and illnesses, that the physicians could follow up. With some exceptions physicians thought the question had improved the consultations and their understanding of the patient.

### Strengths and limitations

This study is based on authentic consultations involving a number of GPs at various healthcare centres in Sweden and patients of widely ranging ages, in diverse life situations, and with different diseases. Topics brought up often concerned delicate personal matters and emotional reactions were common, suggesting that voice-recording had minimal restraining effect on what was said. The doctors were unfamiliar with asking about self-rated health, however, and it was sometimes difficult for them to use the question.

The research material consisted of audiorecordings, verbatim transcripts, and answers to a short questionnaire. For the analysis, STC was used, alongside time measurement and simple counting of yes/no answers in the questionnaire.^14^ The two main themes, reaction and reflection, proved stable during the continuing STC analysis. The doctors’ comments were used to mirror other findings, and also provided information about how the question was experienced by GPs. Altogether, these methods formed parts of a triangulation.

Despite the limited size of the study and the specific Swedish setting, it is believed that the findings are credible and transferable to other settings.

### Comparison with existing literature

The allotment of speaking time between patient and doctor shifted noticeably after the question was asked and more time was used by the patients. This increase is in keeping with the observation that focus was on the patient’s thoughts when the question was posed. Discussion of self-rated health was incorporated within the timeframe of regular consultations at Swedish healthcare centres, which ordinarily last 15–30 minutes.^16^ This is somewhat longer than figures reported in other European countries, ranging from 1 minute to 59 minutes, with a median of 11 minutes.^17^ Longer consultations presumably encompass several significant aspects of a patient’s care.^18^ It has been posited that a question about self-rated health could save time because it helps summarise a great deal of information.^3^

Self-rated health is a relatively stable construct, established early in life.^19^ The patients’ immediate reaction to the question is interpreted as an activation of associations and emotions already at hand. This could account for some of the intense reactions evoked. Many patients made a connection to lifestyle and unhealthy behaviour, usually awakening feelings of failure and shame. The medical profession’s overall emphasis on ‘a healthy lifestyle’ may have influenced their perception of the word ‘health’ and what the doctor was aiming at. The use of strong language and profanity did not otherwise occur during consultations. As swearing can convey anger, grief, and a sense of futility, taking note of these emotions should be a priority in consultations.

In their reflections, patients attempted to understand or explain their spontaneous responses. Sometimes, but not always, this prompted reconsideration of their situation. Opportunities for reflection are few in an ordinary GP consultation, which comprises informational discussion of care and treatment, social chit-chat, or questions and answers.^20^,^16^ The primary significance of such reflection is not to provide information to the doctor, but to provide room for the patients to think through their health situation and perhaps see possibilities or obstacles that may not otherwise be apparent.^21^,^8^ Comparisons with others seemed to stimulate this process. For some patients, however, reflection and comparison strengthened negative feelings about their illness. Physicians generally reported gleaning important information from this reaction, but may initially be at a loss about how to respond.

### Implications for research and practice

The question about comparative self-rated health constitutes a feasible tool in general practice, particularly to solicit information on risk and the patient’s feelings related to an illness or disease, and to encourage the patient’s active reflection on functional abilities, life situation, health, and health strategies. Self-ratings are not to be seen, however, as a standard procedure in all consultations.

There is a need for professional training in using self-rated health questions. Further research is also needed in general practice in different countries about comparative self-rated health and the effects of reflection on health and health care.^22^

## References

[1] Latham K, Peek CW (2013). Self-rated health and morbidity onset among late midlife US adults. J Gerontol B Psychol Sci Soc Sci.

[2] Jylhä M (2009). What is self-rated health and why does it predict mortality? Towards a unified conceptual model. Soc Sci Med.

[3] Undén AL, Elofsson S (2001). Health from the patient’s point of view. How does it relate to the physician’s judgement?. Fam Pract.

[4] Nielsen ABS, Gannik D, Siersma V, Olivarius N de F (2011). The relationship between HbA1c level, symptoms and self-rated health in type 2 diabetic patients. Scand J Prim Health Care.

[5] Wennberg P, Rolandsson O, Jerdén L (2012). Self-rated health and mortality in individuals with diabetes mellitus: prospective cohort study. BMJ Open.

[6] Riddle DL, Dumenci L (2013). Self-rated health and symptomatic knee osteoarthritis over three years: data from a multicenter observational cohort study. Arthritis Care Res (Hoboken).

[7] Siedlecki SL (2006). Predictors of self-rated health in patients with chronic nonmalignant pain. Pain Manage Nurs.

[8] Malterud K, Hollnagel H (2004). Positive self-assessed general health in patients with medical problems. A qualitative study from general practice. Scand J Prim Health Care.

[9] Idler EL, Benyamini Y (1997). Self-rated health and mortality: a review of twenty-seven community studies. J Health Soc Behav.

[10] Baron-Epel O, Kaplan G (2001). General subjective health status or age-related subjective health status: does it make a difference?. Soc Sci Med.

[11] Sargent-Cox KA, Anstey KJ, Luszcz MA (2008). Determinants of self-rated health items with different points of reference: implications for health measurement of older adults. J Aging Health.

[12] Waller G, Thalén P, Janlert U (2012). A cross-sectional and semantic investigation of self-rated health in the northern Sweden MONICA-study. BMC Med Res Methodol.

[13] Roter D, Larson S (2002). The Roter interaction analysis system (RIAS): utility and flexibility for analysis of medical interactions. Patient Educ Couns.

[14] Malterud K (2012). Systematic text condensation: a strategy for qualitative analysis. Scand J Public Health.

[15] Kvale S, Brinkmann S (2008). InterViews: learning the craft of qualitative research interviewing.

[16] Andersson SO, Ferry S, Mattsson B (1993). Factors associated with consultation length and characteristics of short and long consultations. Scand J Prim Health Care.

[17] Deveugele M, Derese A, De Bacquer D (2004). Consultation in general practice: a standard operating procedure?. Patient Educ Couns.

[18] Wilson A, Childs S (2002). The relationship between consultation length, process and outcomes in general practice: a systematic review. Br J Gen Pract.

[19] Vie TL, Hufthammer KO, Holmen TL (2014). Is self-rated health a stable and predictive factor for allostatic load in early adulthood? Findings from the Nord Trøndelag Health Study (HUNT). Soc Sci Med.

[20] Deveugele M, Derese A, De Maeseneer J (2002). Is GP-patient communication related to their perceptions of illness severity, coping and social support?. Soc Sci Med.

[21] Angus L, Levitt H, Hardtke K (1999). The narrative processes coding system: research applications and implications for psychotherapy practice. J Clin Psychol.

[22] Jones P, McDowell A, Viney R (2013). Coaching for health: holding the curtains so patients can change. Br J Gen Pract.

